# Tripartite Motif-Containing 22 Gene -364T/C Polymorphism Associated With Hepatitis B Virus Infection in Chinese Han Population

**DOI:** 10.5812/hepatmon.12110

**Published:** 2014-01-09

**Authors:** Ning Zhao, Xue-Lian Wang, Qiu-Hong Gu, Fen Huang, Wei Zheng, Zhi-Wei Li

**Affiliations:** 1 Department of Infectious Diseases, Shengjing Hospital, Affiliated Hospital of China Medical University, Shenyang, China; 2 Department of Pathophysiology, China Medical University, Shenyang, China

**Keywords:** Hepatitis B virus, TRIM22 Protein, Human, Polymorphism, Single Nucleotide

## Abstract

**Background:**

Hepatitis B virus (HBV) infection significantly contributes to the onset of liver disease and hepatocellular carcinoma. Understanding the pathogenesis of HBV infection susceptibility could help us to control HBV infection effectively.

**Objectives:**

This study investigated single nucleotide polymorphisms (SNPs) of the tripartite motif-containing 22 (TRIM22) gene associated with HBV infection outcome.

**Patients and Methods:**

A total of 765 Chinese Han subjects were enrolled: 293 patients were presented with chronic hepatitis B (CHB), 224 were asymptomatic HBV carriers, 248 had self-limited HBV infection, and all of them were recruited for TRIM22 SNPs genotyping. RING and SPRY domains of TRIM22 gene were DNA-sequenced, and HBV serum markers and HBV DNA were measured quantitatively in all subjects.

**Results:**

243 (31.76%) of 765 Chinese Han patients showed genetic variation in the TRIM22 gene. TRIM22 SNPs were mainly in RING area -364T/C site, accounting for 98.35% of the population. There were no significant differences (P > 0.05) in the RING domain -364T/C SNP and allele frequencies between patients with chronic hepatitis and asymptomatic HBV carriers. The CC genotype of TRIM22 gene RING domain -364T/C locus (rs10838543) was associated with chronic HBV infection (OR = 2.30, 95% CI = 1.24-3.97, P = 0.0012; OR = 2.26, 95% CI = 1.08-3.74, P = 0.002) and a mutant allele C carrier of the TRIM22 gene was associated with HBV chronic infection (OR = 1.97, 95% CI = 1.10-3.75, P = 0.0049; OR = 2.12, 95% CI = 1.17-3.89, P = 0.0038).

**Conclusions:**

TRIM22 gene RING domain -364T/C polymorphism is associated with chronic HBV infection in Chinese Han population.

## 1. Background

Hepatitis B virus (HBV) infection is a worldwide health issue, causing the onset of acute and chronic hepatitis, cirrhosis, and hepatocellular carcinoma. Although the HBV infection is a serious burden to human health, outcomes of different HBV infected subjects are different; for example, approximately 90% of the adults, acutely infected with HBV, can spontaneously eliminate the virus (1); whereas, 5% -10% of the infected subjects would develop a chronic infection, with approximately 5% of patients progressing to cirrhosis and even hepatocellular carcinoma (2). Patients' characteristics (such as age at infection and virus load) and the level of the immune response against HBV infection may affect the outcome of HBV infection, in addition to certain gene expressions and single nucleotide polymorphisms (SNPs). A previous study showed that the polymorphisms of the tumor necrosis factor-alpha gene promoter region impacted the course of spontaneous HBsAg clearance in HBV-infected patients (3). Thus, further studies on different genes and their SNPs could gather useful information for better understanding/characterizing HBV infection susceptibility and liver diseases. 

To this end, tripartite motif proteins (TRIM) are a superfamily of proteins combining three structural domains of RING, B-box, and coiled-coil region. TRIM protein is involved in a variety of physiological cell activities, such as cell growth, inhibition, or apoptosis (4-6). TRIM family proteins (like TRIM5α, TRIM19, and TRIM25) play an important role in inherent antiviral immunity (7-9), while TRIM22 protein is localized in the cytoplasm, its expression is interferon- induced, and is also a target gene for the tumor suppressor gene p53 (10). Recent studies have shown that TRIM22 protein inhibits HBV gene expression and replication in a dependent manner effectively by suppressing HBV core promoter activity in RING domain. The SPRY domain drives/leads TRIM22 protein nuclear localization, and the RING domain inhibits the activity of four HBV gene promoters, especially the S and core promoters. Induction of TRIM22 protein weakens HBV gene expression and DNA replication ultimately, and thus, reduces hepatitis B virus surface antigen (HBsAg) and hepatitis B e antigen (HBeAg) expression (11).

Thus, detection of TRIM22 gene polymorphisms could help us to predict HBV infection susceptibility and HBV-induced diseases outcome. TRIM22 gene is localized in chromosome 11p15 (12). This study investigated whether the polymorphisms of SPRY and RING domains of TRIM22 gene could affect patient immune response to HBV infection, resulting in different clinical outcomes in a HBV-infected Chinese Han population.

## 2. Objectives

This study investigated single nucleotide polymorphisms (SNPs) of TRIM22 gene associated with HBV infection outcome.

## 3. Patients and Methods

### 3.1. Study Population

Patients were enrolled between August 2008 and October 2011 in our institution and participated to our epidemiological survey. They included 293 patients with chronic hepatitis B (CHB), 224 asymptomatic HBV carriers (AsC), and 248 patients with self-limited HBV infection (SL) (Table 1). All patients were residents of the Liaoning province, China. 

**Table 1.** Demographics and Environmental Risk Factors ^a^

	CHB ^b^(n = 293)	AsC ^b^ (n = 224)	SL ^b^ (n = 248)	CHB vs. AsC, P value	CHB vs. SL, P value	AsC vs. SL, P value
**Age, mean ± SD, y**	46.35 ± 8.67	32.66 ± 7.32	40.81 ± 9.17	0.006	0.14	0.08
**Gender, Male/Female, No.**	210/83	138/86	116/132	0.0062	0.0007	0.0045
**Smoker, No. (%)**	83 (28.30)	50 (22.30)	63 (25.40)	0.107	0.26	0.23
**Alcohol drinker, No. (%)**	95 (32.42)	28 (12.50)	69 (27.82)	0.0003	0.34	0.0007
**ALT ^b^, mean ± SD , U/L**	207.12 ± 70.02	20.34 ± 6.21	19.56 ± 5.54	8.91E-5	2.24E-6	0.53
**AST ^b^, mean ± SD, U/L**	165.33 ± 52.26	17.87 ± 4.76	17.03 ± 5.01	4.77E-5	5.83E-5	0.49
**HBeAg positive, No. (%)**	128 (43.69)	108 (48.21)	0	0.090		
**HBVDNA, mean **± **SD, log10 IU/ml**	5.43 ± 1.18	6.06 ± 1.24	≤ 1.08	0.130		

^a^ ALT, M: <45, F: < 34; AST, M: < 35, F: < 31; HBV DNA, ≤ 12 IU/ml.

^b^ Abbreviations: ALT, alanine aminotransferase; AsC, asymptomatic HBV carriers; AST, aspartate aminotransferase; CHB, chronic hepatitis B; SL, self-limited HBV infection.

The diagnostic criteria for asymptomatic HBV carriers were serum HBsAg positivity for over one year, normal liver function tests, and no clinical symptoms. Conversely, patients presenting persistent abnormal serum glutamate aminotransferase (ALT) values or aspartate aminotransferase (AST) levels (twice the level of the upper limit), or liver histological examination, were diagnosed as CHB. Self-limited HBV infection was defined as positive results for serum anti-HBs and/or anti-HBc, but negative findings for HBV DNA, and no history of hepatitis B vaccination. The exclusion criteria were: 1) Non-HBV-related acute/chronic hepatitis; 2) Liver cirrhosis or hepatocellular carcinoma; 3) Non-Chinese Han subjects; 4) Mother to child transmission; 5) Patients presenting HBsAg seroconversion post-CHB treatment. This study was approved by the Ethics Committee of China Medical University and complied with the ethical guidelines of the Declaration of Helsinki 1975. A written informed consent was collected from each patient. 

### 3.2. Hepatitis B Virus Infection Detection

Serum levels of HBsAg, anti-HBs, HBeAg, anti-HBe, and anti-HBc were tested by a chemiluminescence assay (Abbott Laboratories, Delkenheim, Germany). 

### 3.3. HBV DNA Quantitative Detection 

HBV DNA quantitative detection was assessed by COBAS AmpliPrep/ COBAS TaqMan 48 Analyzer (Roche Molecular Systems, Branchburg, NJ, the USA) with the HBV nucleic acid quantification detection kit according to the manufacturer’s instructions (Roche Molecular Systems).

### 3.4. TRIM22 Gene Genotyping

Human genomic DNA was extracted from peripheral blood leukocytes using a DNA extraction kit (Tiangen Biotech Co., Ltd., Beijing, China), and then analyzed by PCR for TRIM22 RING and SPRY domains. The PCR products were then sequenced and analyzed by ABI 3700 DNA automated sequencer (Applied Biosystems, Foster City, CA). Primers of the TRIM22 gene RING domain were: 5'-ATGATGGGTTACACGAAGC-3' and 5'-ATTGCCACTCTTCCCACAT-3'. Primers of TRIM22 gene SPRY domain were: 5'-TGTCTCTTCTCAATGCTGTA-3' and 5'-CAATGTGAAGAGTCATAGGGA-3'. Moreover, DNA sequencing primer of TRIM22 gene RING domain was 5'-AAGCAGTATTCTTTCTATTC-3', and DNA sequencing primer of TRIM22 gene SPRY domain was 5'-TGTCTCTTCTCAATGCTGTA-3'. 

### 3.5. Statistical Analysis

Hardy-Weinberg genetic equilibrium law test was performed using SHEsis software (http://analysis2.bio-x.cn/myAnalysis.php). Differences in group allele frequency and genotype frequency were analyzed by 2×2 or 2×3 tables using chi-square test. A multivariate unconditional logistic regression analysis was performed to calculate odds ratios (OR), 95% confidence intervals (CI) were used to evaluate the association between SNPs genotype and HBV infection. Statistical analysis was performed by means of SAS9.1 statistical analysis package (SAS Institute Inc., Cary, NC, the USA). All statistical tests were two-sided, and P < 0.05 was considered statistically significant. 

## 4. Results

### 4.1. Sequencing of the RING and SPRY Domains of TRIM22 Gene 

**Figure 1.**
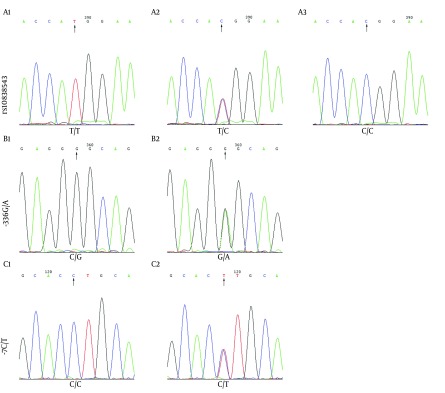
Representative DNA Sequencing Data on the RING and SPRY Domains of TRIM22 Gene A, There were TT, TC and CC genotypes in RING area-364T/C site. B, there were GG and GA genotypes in RING area-336G/A site. C, there were CC and CT genotypes in SPRY domain-7C/T site.

Two hundred and forty three (31.76%) of 765 Chinese Han patients were confirmed as presenting genetic variations in the RING and SPRY domains of TRIM22 gene. Overall, three types of gene mutations were detected: 2 in RING-336G/A locus, 239 in RING area-364T/C site, and 3 in SPRY domain-7C/T site. One case presented both -336G/A and -364T/C site genetic variations. SNPs mainly occurred in RING area-364T/C site, accounting for 98.35% of all SNPs detected in this study population. There were TT, TC and CC genotypes in -364T/C site, GG and GA genotypes in -336G/A site, CC and CT genotypes in -7C/T site (Figure 1). 

### 4.2. TRIM22 Polymorphism Association in Chinese Han HBV-Infected Population 

TRIM22 gene RING domain -364T/C SNP genotype and allele frequency distribution in self-limiting infection subjects, asymptomatic HBV carriers and patients with chronic hepatitis are shown in Table 1. SNPs genotype frequency distribution in HBsAg carrier and self-limited infection groups are in line with the Hardy-Weinberg genetic equilibrium (P > 0.05). 

As shown in Table 2, there are no significant differences (P > 0.05) in the RING domain SNP -364T/C genotype and allele frequencies between patients with chronic hepatitis and asymptomatic HBV carriers. However, patients with chronic hepatitis of -364T/C locus CC genotype frequency was significantly higher than that of the self-limiting infection subjects (P = 0.004), asymptomatic HBV carriers -364T/C locus CC genotype frequency was significantly higher than that of the self-limiting infection subjects (P = 0.009). Chronic hepatitis -364T/C sites C allele frequency was significantly higher than that of the self-limiting infection subjects (P = 0.02), asymptomatic HBV carriers -364T/C sites C allele frequency was significantly higher than that of the self-limiting infection subjects (P = 0.03).

**Table 2.** Genotype Distribution and Allele Frequencies of TRIM22 SNPs in the Different Population Groups

-364 T/C	CHB ^a^, No. (%)	AsC ^a^, No. (%)	SL ^a^, No. (%)	CHB vs. AsC		CHB vs. SL		AsC vs. SL	
				χ^2^	P value	χ^2^	P value	χ^2^	P value
**Genotypes**				0.28	0.88	10.22	0.004	9.27	0.009
TT	182 (62.11)	146 (65.18)	198 (79.84)						
TC	49 (16.72)	38 (16.96)	26 (10.48)						
CC	62 (21.17)	40 (17.86)	24 (9.68)						
**Alleles**				0.32	0.57	5.14	0.02	4.86	0.03
T	413 (70.47)	330 (73.66)	422 (85.08)						
C	173 (29.53)	118 (26.34)	74 (14.92)						

^a^Abbreviations: AsC, asymptomatic HBV carriers; CHB, chronic hepatitis B; SL, self-limited HBV infection.

If self-limited HBV infection subjects were recruited as the control group, multivariate logistic regression analysis showed that TRIM22 -364T/C SNP CC genotype was associated with chronic HBV infection (OR = 2.30, 95% CI = 1.24-3.97, P = 0.0012; OR = 2.26, 95% CI = 1.08-3.74, P = 0.002). DNA samples carrying at least a mutant allele C were statistically associated with chronic HBV infection (OR = 1.97, 95% CI = 1.10-3.75, P = 0.0049; OR = 2.12, 95% CI = 1.17-3.89, P = 0.0038) (Table 3).

**Table 3.** Association of TRIM22 SNPs in the Studied Population Groups

Genotypes	CHB ^a^, No. (%)	AsC ^a^, No. (%)	SL ^a^, No. (%)	CHB vs. AsC		CHB vs. SL		AsC vs. SL	
				OR (95% CI)	P value	OR (95% CI)	P value	OR (95% CI)	P value
**TT**	182 (62.11)	146 (65.18)	198 (79.84)						
**TC**	49 (16.72)	38 (16.96)	26 (10.48)	1.03 (0.53-1.59)	0.58	1.67 (1.08-2.85)	0.018	1.98 (1.25-3.01)	0.012
**CC**	62 (21.17)	40 (17.86)	24 (9.68)	1.24 (0.89-2.08)	0.31	2.30 (1.24-3.97)	0.0012	2.26 (1.08-3.74)	0.002
**TC+CC**	111 (37.89)	78 (34.82)	50 (20.16)	1.14 (0.77-1.58)	0.39	1.97 (1.10-3.75)	0.0049	2.12 (1.17-3.89)	0.0038

^a^Abbreviations: AsC, asymptomatic HBV carriers; CHB, chronic hepatitis B; SL, self-limited HBV infection.

## 5. Discussion

Chronic HBV infection is related to several factors with age as the most influencing one. Respectively 90% of HBV mother-to-child infected (perinatal period) patients, 25%~30% HBV childhood-infected patients, and 5%-10% HBV patients, who contracted HBV after 5 years of age, would develop a chronic infection (13). However, even if infected in the same age of host, the outcomes of HBV infection may still be different. To eliminate the impact of perinatal HBV transmission on the chronic HBV infection outcome, those particular subjects were excluded from the study. Moreover, a previous study showed that a person infected by HBV C genotype had less potential to progress to chronic hepatitis (14); whereas, a European study demonstrated that HBV A genotype infection was easily progressing to chronic infection versus HBV genotype D (15). In addition, clinical studies also revealed that the efficacy and clinical outcomes of both acute and chronic hepatitis B infections were not the same, even when the same treatment was used (16). 

Furthermore, differing individual responses to HBV infection are also affected by the host genetic factors. For example, expression of human leukocyte antigens (HLA) allele gene can affect HBV infection outcomes (17). Many scholars have systematically studied the correlation between HLA allele polymorphism and HBV infection, and found that the HLA-A*33, B*08, B*44, and B*35 gene alleles were associated with persistent HBV infection (18, 19), and the HLA-A*0301, A*02 and B*4001 gene alleles were associated with HBV viral clearance (20-22). Further haplotype analyses showed that haplotype containing HLA-I molecules B*44, such as B*44-DR7, B*44-DRB1*0701, B*44-Cw*1601-DRB1*0701, and A33-DR7 were associated with persistent HBV infection (19, 21). In addition, polymorphisms of genes encoding a variety of cytokines such as interferon (IFN), interleukin (IL), tumor necrosis factor (TNF), and other cytokines, could also be associated with different HBV infection outcomes. Expression of these genes may play a role in the regulation of the host immune responses to HBV infection and lead to independent virus clearance or persistent HBV infection (23-25). Indeed, more and more studies have recently shown that innate immune genes and their expressions play an important role in regulation of antivirus infection.

Our current study was the first to investigate whether the TRIM22 SNPs affects HBV infection outcome. TRIM family proteins have been shown to regulate antiviral innate immune responses. TRIM22 protein has recently been revealed to inhibit HBV infection in humans. In HBV infection, TRIM22 protein would be activated, and subsequently, the appropriate immune response would be induced and inhibit HBV replication, leading to HBV clearance (12). Thus, in the current study, we genotyped the coding region of TRIM22 RING and SPRY domains in 765 Chinese Han patients. The data showed that TRIM22 SNPs occurred in 31.76% of these subjects, and SNP locus was mainly localized in -364T/C site. CC genotype of TRIM22 gene RING domain -364T/C locus (rs10838543) was associated with chronic HBV infection, and a mutant allele C carrier of TRIM22 gene was associated with HBV chronic infection. These data are novel and to date, there is only limited or no study on TRIM22 gene SNP distributions association with HBV infection, although SPNs in other genes were associated with susceptibility to HBV and clinical outcomes of HBV infection in different ethnic populations (26-28). 

The SNPs are the most common genetic variations in human genome. Certain SNPs would alter protein coding; whereas others, like synonymous SNPs, alter codon composition but do not change the encoded amino acid, which is called silence SNPs, which is often overlooked in associated studies. However, recent studies showed that some synonymous SNPs are related to disease risk, while others affect the efficacy of drug action. For example, the synonymous SNPs of corneodesmosin was associated with psoriasis onset (29), the synonymous SNPs of ApoE gene and low-density lipoprotein receptor-related protein 6 gene can increase the risk in developing Alzheimer's disease (30, 31). The synonymous SNPs affect protein translation speed and expression while affecting translation efficiency, mRNA stability and splicing controls (32-35). Study of the synonymous SNPs on multidrug resistance gene 1 (MDR1) led to the discovery of new mechanisms responsible for protein folding kinetics control, causing protein local fine structural changes, affecting the binding specificity and affinity to drugs (36). Thus, further studies could investigate previously ignored synonymous SNPs in gene polymorphism screening and molecular markers identification to assess their potential function in human body. TRIM22 -364T/C SNP did not alter the encoded amino acid, but it did affect HBV infection outcome. 

In conclusion, our current study demonstrated that TRIM22 gene RING domain -364T/C SNP was associated with chronic persistent HBV infection in Han Chinese population. Further studies are recommended to investigate underlying molecular mechanisms better.
